# SARS-CoV-2 Infection and Taste Alteration: An Overview

**DOI:** 10.3390/life12050690

**Published:** 2022-05-06

**Authors:** Gaetano Scotto, Vincenzina Fazio, Eleonora Lo Muzio, Lorenzo Lo Muzio, Francesca Spirito

**Affiliations:** 1 Infectious Diseases Unit, University Hospital “OORR” Foggia, 71122 Foggia, Italy; gaetano.scotto@unifg.it; 2 Department of Prevention, Hygiene and Public Health Unit, University Hospital “OORR” Foggia, 71122 Foggia, Italy; vincenzina.fazio@unifg.it; 3 Department of Dental Sciences, University of Ferrara, 44121 Ferrara, Italy; eleonoralomuzio@gmail.com; 4 Department of Clinical and Experimental Medicine, University of Foggia, 71122 Foggia, Italy; spirito.francesca97@gmail.com

**Keywords:** COVID-19, SARS-CoV-2, ageusia, dysgeusia, taste, gustatory dysfunction

## Abstract

Since the worldwide spread of SARS-CoV-2 infection, the management of COVID-19 has been a challenge for healthcare professionals. Although the respiratory system has primarily been affected with symptoms ranging from mild pneumonia to acute respiratory distress syndrome, other organs or systems have also been targets of the virus. The mouth represents an important route of entry for SARS-CoV-2. Cells in the oral epithelium, taste buds, and minor and major salivary glands express cellular entry factors for the virus, such as ACE2, TMPRSS2 and Furin. This leads to symptoms such as deterioration of taste, salivary dysfunction, mucosal ulcers, before systemic manifestation of the disease. In this review we report and discuss the prevalence and socio-demographics of taste disturbances in COVID-19 patients, analysing the current international data. Importantly, we also take stock of the various hypothesized pathogenetic mechanisms and their impact on the reported symptoms. The literature indicated that COVID-19 patients frequently present with gustatory dysfunction, whose prevalence varies by country, age and sex. Furthermore, this dysfunction also has a variable duration in relation to the severity of the disease. The pathogenetic action is intricately linked to viral action which can be expressed in several ways. However, in many cases these are only hypotheses that need further confirmation.

## 1. Introduction

**Figure 1:**
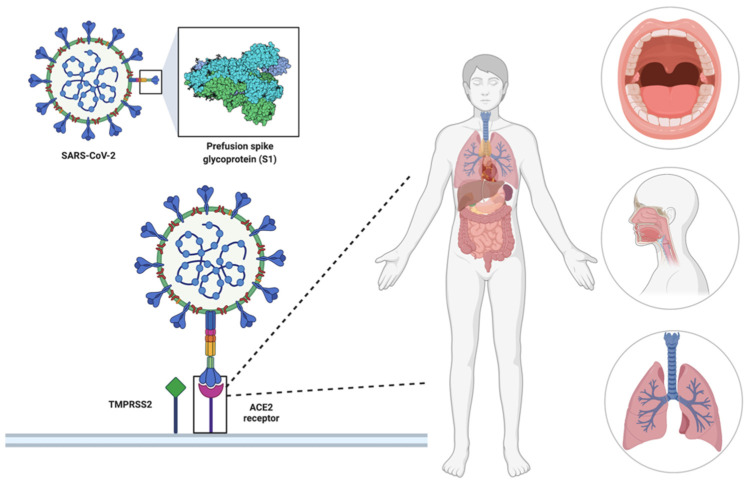
Mechanism of entry of the virus into the host cells through the binding between the spike glycoprotein and the ACE2 receptor, expressed in several organs and systems of the human body. Created with BioRender.com (accessed on 9 March 2022).

Since January 2020, the spread of the new highly infectious SARS-CoV-2 virus and the evidence of new viral variants has been an international public health problem. The state of emergency arises from the high infectivity, morbidity, mortality together with an increasingly varied symptomatology and different prognostic factors. The clinical evolution as well as the pathogenetic mechanism are yet to be fully characterized. To date, given the absence of a proven effective drug therapy, an extensive vaccination campaign seems to be the best strategy to contain the spread of the SARS-CoV-2 virus [1]. The clinical picture of SARS-CoV-2 infection is highly variable, ranging from asymptomatic or medium severity, involving the upper respiratory tract, more severe involving both lungs to critical respiratory distress. The most common symptom picture of coronavirus disease-19 (COVID-19) includes fever, fatigue, dry cough often accompanied by sputum production, headache, haemoptysis, anorexia, sore throat [2]. In addition to the clinical scenario described above, a variety of other “non-typical” manifestations have been observed, including neurological, psychiatric and neuropsychiatric, cardiovascular, gastrointestinal and dermatological, as an expression of coronavirus SARS-CoV-2 trophism for more human tissues [3]. The particular multi-organ tropism that characterizes the this coronavirus is linked to the mechanism of penetration of the virus into the host cells which occurs through the bond between the homotrimeric spike glycoprotein present on the virion surface and the angiotensin converting enzyme 2 (ACE2) receptor, which is represented in different organs and systems of the human body [4]. Amongst the extrapulmonary manifestations the high prevalence, albeit belatedly identified, of olfactory (anosmia) and gustatory (ageusia, dysgeusia) dysfunctions has attracted a lot of attention, due to the subjective peculiarity of the symptoms [5]. This has now become a sort of “distinctive trademark” of the disease. The mouth, as well as the nasal cavities, represents an important route of entry for SARS-CoV-2: the epithelial cells of the dorsal surface of the tongue, the taste buds, especially the fungiform ones, major and minor salivary glands express a high concentration of cellular entry factors for SARS-CoV-2, such as ACE2 receptor [6], transmembrane protease serine 2 (TMPRSS2) and Furin [7,8]. SARS-CoV-2 enters target cells mainly through the interaction between the viral spike protein (protein S) and the ACE2 receptor [6,9]. In addition, the virus uses the TMPRSS2 for protein S priming [7] (Figure 1). These findings suggest that the mucosa of the oral cavity may be a potentially high-risk pathway for SARS-CoV-2 infection and that the replicating virus in the infected host cell could frequently determine regional pathologies in the form of ulcerative lesions, together with vesicles and blisters, petechiae, and erythema multiforme-like lesions, aphthous-like lesions, herpetiform lesions and symptoms such as taste alteration [10], but could also transmit to other organs or systems.

In this review we will deal with the epidemiological, pathogenetic and clinical aspects of taste alteration (ageusia, hypogeusia, dysgeusia) in patients with COVID-19.

## 2. Demographic and Epidemiological Characteristics

Data obtained from both systematic reviews of articles and meta-analyses showed that the prevalence of altered sense of taste varied from study to study ranging from 1.0% to 93.0% [11,12,13,14]. High variability may depend on or be related to sex, age, or finally, to the country of origin or ethnicity, but also to differences in research methods [15].

The alteration of taste associated with COVID-19 seems to be related to gender even if the question remains controversial; according to some studies, females manifest taste dysfunction with a prevalence of 52.6–57.1% of cases, higher compared to males who manifest this symptom with a prevalence of 25.0–36.5% [16,17]. Other studies have confirmed a higher prevalence of dysgeusia in females compared to males in both European and Asian countries [18,19,20,21], such as a study conducted in Korea in which data about anosmia and ageusia symptoms among 3191 patients were collected and whose results showed a higher prevalence in females (68.9%) than males (31.1%) [18]. A European confirmation of this data derives from the results of a multicentre study in which sex-based analysis comparing the occurrence of olfactory and gustatory dysfunctions between men and women was performed and that showed that females were significantly more affected by these symptoms than males [22]. However, other authors have a completely different observation, and do not find any correlation between dysgeusia and sex [23]. In fact, it has been observed that the expression of ACE2 and TMPRSS2 is superimposable in the two sexes [24]; Based on this evidence one might expect that there can be no gender diversity with regard to dysgeusia [25], but there are differential pathological mechanisms that could explain it. It is assumed, for example, that the higher prevalence of taste disturbances amongst women could be linked to differences in the perception of taste between the two sexes, with females having greater reactivity to gustatory stimuli than males [26]. This may be a consequence of the higher number of fungiform papillae and taste buds that women have compared to men. Thus, the gender difference in taste perception could be associated with the different prevalence of gustatory dysfunction [27].

Regarding age distribution, in general, children appear less susceptible to SARS-CoV-2 infection, presenting fewer and milder symptoms [28]. The prevalence of olfactory and gustatory dysfunctions seems not to be high and, certainly, lower than that of adults [29] although the data are conflicting and reports of these symptoms in young people are increasing. In a study conducted in France it has been demonstrated a 1% prevalence of ageusia in cohort of primary school children [30]. Indeed, when assessing the child/adult prevalence ratio in a cohort of Israeli patients, children had a prevalence index of 25.8% compared to 71.4% in adults [31]. However, the results of other studies show that this symptom is not so uncommon in the young population affected by COVID-19, as demonstrated by an international multicentric study that showed a prevalence of these symptoms of 37% in children [32]. Another study conducted in Korea also shows how this symptom is manifesting more and more frequently in young patients [18]. In general, in almost all reported paediatric cases, gustatory disturbances were accompanied by mild or moderate disease [32] and sometimes dysgeusia was the only symptom [33].

Epidemiological studies on the geographical differences, have indicated that the prevalence of gustatory dysfunction is extremely variable, and is linked to the country of origin and to different ethnic groups. In a systematic review including over 10,000 COVID-19 patients, 53% of North American patients, 50% of European patients, and 27% of Asian patients had taste disturbances [34]. This diversity of prevalence was further confirmed by another study, in which taste dysfunctions were observed more frequently in European cohorts (34–86%) [albeit with differences within the same continent (Germany 69.2%, France 49.1%)], in North America (19–71%) and the Middle East (36–98%) compared to Asian cohorts (11–15%) [35]. As for ethnicity, a meta-analysis study showed that dysgeusia is six times more frequent in Caucasians than in Far East Asians [13]. This interesting observation is confirmed by an Italian meta-analysis publication, which analysed 67 studies including 27,687 cases of COVID-19 from 16 countries, and found that the prevalence of dysgeusia ranged between 5.6% and 96% in relation to the different geographical areas [36] These differences could be due to genetic variations related to the ACE2 receptor and the TMPRSS2 enzyme. In fact, a comparative genetic analysis of SARS-CoV-2 suggested that the expression of ACE2 receptor and TMPRSS2 differs between different ethnic groups, and between Asian and European populations as the genes that code for these proteins vary depending on the country or ethnicity [37,38]. In particular the East Asian populations seems to have much higher occurrence of variants associated with higher ACE2 receptor expression in tissues, which may suggest the development of a different symptomatologic picture in response to SARS-CoV-2 infection [37].

## 3. Physiological Aspects of Taste

In humans, the taste system includes taste cells, afferent gustatory nerves and brain structures involved in the central processing of taste. The process begins with taste receptor cells organized into taste buds, most of which are located on the dorsal surface of the tongue. In each taste buds there are 150 to 300 epithelial cylindrical cells and five cell types: dark-toned cells (type I cells) occupy 50–70% of the taste buds; light-toned cells (type II cells) occupy 15–30% of the taste buds; taste cells (type III) occupy 5–15% of the taste buds; type IV cells (basal cells) are present at the base of the taste buds [24,38]; and type V cells (marginal cells); in addition there are also neuronal fibres that after penetrating the basal membrane, form an unmyelinated plexus within the taste bud [39]. Type I cells are glial-like cells with several long microvilli and are involved in salty taste recognition [40]; Type II cells are responsible to mediate sweet, umami and bitter tastes through the interaction between the GPCR (g-protein-coupled receptor) receptors and the external molecules. Type III cells are presynaptic cells thought to mediate sour and salty taste and are responsible to transmit information from the type II cell through P2Y adenosine receptors, and mediate signals transmission to the afferent neurons by releasing serotonin [41]. Finally, type IV are precursor cells that differentiate into the cell types previously described, allowing for a rapid cell turnover of the gustative buds [42]. So, types I-III are specialized cells with taste receptor function and are exposed in the oral cavity to interact with taste stimuli via taste receptor proteins. This interaction results in a signal that is transmitted through the gustatory nerves afferent to the brain to evoke the perception of taste. Although a large number of chemicals can be perceived, there are currently five different taste qualities: bitter, sweet, sour, salty and umami [43]. The perception of different tastes occurs thanks to receptors present on the surface of specialized epithelial cells (called TRCs or taste receptor cells) which are found inside the gustatory calyxes, mainly located on the tongue [44]. Each taste quality is detected through a different molecular mechanism of transduction. In particular, salty and acid act directly on membrane ion channels, while sweet, umami and bitter use transduction mechanisms mediated by taste receptors associated with G proteins (GPCR) [45]. The main receptor for the salty taste is an epithelial amiloride sensitive Na^+^ channel called epithelial sodium channel (ENaC) [46]. There are numerous genes that code for bitter taste receptors. In particular, a family of 25 genes, called T2Rs or TAS2Rs, were identified in humans, expressed separately in type II cells in the taste buds [33] and located on chromosomes 12, 7 and 5 [47]. Sweet and umami tastes are driven by receptors belonging to the TAS1R family (TAS1R1, TAS1R2, and TAS1R3), which are encoded by the Tas1r genes. In particular, the receptor for the sweet taste is constituted of a dimer formed by TAS1R2 and TAS1R3 [47], while TAS1R3 combined with TAS1R1 forms the dimer responsible for the perception of umami taste [48]. Three nerve pathways are involved in the transmission of information relating to taste perception to the central nervous system. more precisely to the gustatory cortex: the chorda tympani, branch of the facial nerve (VII cranial nerve), the glossopharyngeal nerve (IX cranial nerve), and the vagus nerve (X cranial nerve) [39].

## 4. Pathogenetic Hypotheses of Taste Alteration

The pathogenesis of taste alteration is, at the moment, far from clear. Various hypotheses have been advanced, with sometimes conflicting data, without reaching a definite conclusion.

The presence of both the ACE2 receptor and TMPRSS2 have been widely reported in the taste buds of the tongue. Some Japanese studies [49,50] have indicated that they are constantly expressed and localized in human fungiform taste buds. Observing the unicellular profiles of the tissues of the tongue with typical gene markers of taste buds, such as TAS1R3, TAS2R4, TAS2R14, NCAM1 and SNAP25, it was also shown that the distribution of ACE2-positive cells correlated to that of the cells labelled with genes related to taste [51]. In contrast, another study sequencing the RNA in the epithelial cells of the adult mouse tongue, demonstrated the expression of ACE2 in the basal region of the filiform non-gustatory papillae, but not in the gustatory ones. Therefore, it is implausible that direct infection of taste bud cells by SARS-CoV-2 would cause taste alteration [52]. Instead, the virus would invade the stratified squamous epithelium of the lingual dorsum expressing ACE2, and the filiform papillae close to the taste buds and, therefore, the infection would progress in the taste cells, through a possible cell-cell viral transmission. The latter could occur via the actin-myosin (nanotubes-TNT) system, used by many viruses for transmission from infected cells to neighbouring cells, thus explaining why taste cells that do not express ACE2 are infected through neighbouring ACE2-positive cells [52].

The direct or indirect replication of SARS-CoV-2 in infected taste cells can generate inflammation and cell death, thus causing malfunction of the gustatory system and consequently gustatory dysfunction [53]. The inflammatory response, which is mediated by the interaction of the virus with toll-like receptors, stimulates pro-inflammatory cytokines that trigger the inflammatory process and can also lead to apoptotic cell death, causing an abnormal turnover of the taste buds and, therefore, taste disturbances and possible tissue hypoxia [54]. Taste papillae cells would express cytokine signalling pathways through which inflammation would affect taste function [55].

Furthermore, SARS-CoV-2 can bind to sialic acid receptors, occupying sialic acid binding sites in the taste buds and leading to an early degradation of gustatory molecules. Sialic acid is a fundamental component of salivary mucin and has a protective action on glycoproteins that transport the taste molecules inside the taste pores, protecting them from premature enzymatic degradation [56]. A reduced concentration of salivary sialic acid is associated with an increase in the taste threshold. In doing so, SARS-CoV-2 could then occupy the sialic acid binding sites on the taste buds, accelerating the degradation of taste molecules [57].

SARS-CoV-2’s impact on the nervous system would constitute a further mechanism of action of the virus in the pathogenesis of the alteration of the sense of taste. The perception of flavours is complex and involves the senses of taste and smell [57]. In fact, the sense of taste requires the activation of the gustatory receptors on the tongue, which receive innervation from the cranial nerves VII, IX and X, to recognize the five types of taste: sweet, bitter, salty, acid and umami [55]. The gustatory disturbances would be influenced by the concomitant presence of olfactory disturbances, due to the intimate correlation between these two chemo-sensory systems [58]. Since SARS-CoV-2 targets the nerves of the PNS, direct damage to any of the cranial nerves involved in the transmission of the gustatory stimulus may induce taste dysfunction. One of the proposed pathogenetic mechanisms is that once SARS-CoV-2 enters the oral epithelia through ACE2 and TMPRSS2 to damage taste receptor cells, it then infiltrates the CNS [59,60]. Neuro-invasion by SARS-CoV-2 can occur at the neural-mucosal interface via trans-mucosal entry through regional nerve structures [59]. This may be the underlying mechanism for the infection of taste neuronal fibres in taste buds and the subsequent alterations in taste perception [59]. It has also been hypothesized that SARS-CoV-2 may degrade the CNS by stimulating autoimmune reactions mediated by T lymphocytes towards CNS antigens, thus altering gustatory function [59,61]. Flavour perceptions, however, are combined with those provided by the retronasal olfactory cells to give rise to flavours. This is because most flavours are perceived through the nose rather than the tongue; the flavours spread in the oral cavity and produce a mixed sensation of gustatory and olfactory perception, responsible for the widest perception of taste. The loss of flavour perception would therefore be a natural consequence of the loss of smell [62].

## 5. Host-Related Pathogenetic Factors

### 5.1. Zinc

Zinc is found in high concentrations in taste buds where it plays a significant role in the perception of taste and in the nerve transmission of the gustatory stimulus [63]. Although its role in dysgeusia is little known, it is hypothesized to be a cofactor for alkaline phosphatase in the taste buds, thus making it necessary also for the regeneration and maintenance of taste [64]. Furthermore, zinc could influence the concentration of gustine (zinc-dependent enzyme), through the direct or indirect interaction of carbonic anhydrase-6, which can influence the production of the taste buds [65]. Therefore, it would appear that zinc deficiency can induce changes in the number, size and structure of taste bud cells, as well as a decrease in nerve sensitivity [64,65]. Thus, it is possible that zinc deficiency reduces the rate of proliferation and regeneration of taste buds [63,64]. Confirmation of this hypothesis comes from the evidence that a significant number of COVID-19 patients with worse clinical picture were zinc deficient [66,67].

### 5.2. UGT2A1 and UGT2A2

As previously pointed out, anosmia and dysgeusia are two symptoms that are often interrelated and can occur at the same time. According to a study recently published in Nature Genetics, involving 69,841 people residing in the United States and the United Kingdom (68% of them reported loss of smell or altered taste), it seems that there is a genetic predisposition to influence the probability of encountering these two neurological symptoms; two genes—UGT2A1 and UGT2A2—have been identified as responsible, since both are associated to an 11% increase in chance to develop both anosmia and dysgeusia [68].

UGT2A1 and UGT2A2 are part of the uridine diphosphate glycosyl-transferase family—enzymes that metabolize lipophilic substrates [69]. These molecules are expressed in the olfactory epithelium and play an important role in metabolizing olfactory particles that enter the nasal cavity and transport them to olfactory receptors. If their action is blocked due to SARS-CoV-2, the olfactory bulb is no longer stimulated. This prevents smell from being detected by the brain and the lack of activation creates a genetic link to the bio-mechanisms underlying the loss of smell or taste [70]. In practice SARS-CoV-2 enters the airways and accumulates in the olfactory support cells, in particular in the sustentacular cells which, in contrast with neurons, abundantly express ACE2. These support cells are metabolically and functionally associated with olfactory neurons and with the transmission of the odour signal [71]. Therefore, when their function is interrupted, olfaction is seriously compromised. The loss of smell and, consequently, the loss of flavour perception is, therefore, correlated to the damage caused by the virus to the support cells, not to the infection of the olfactory neurons as one might mistakenly believe [72].

## 6. Clinical Aspects

**Figure 2:**
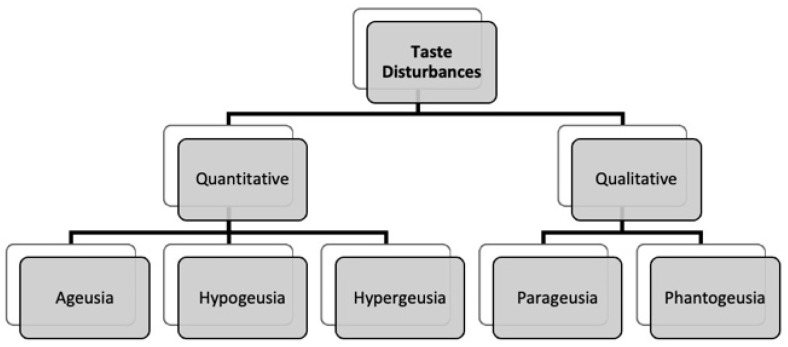
Classification of taste disturbances.

The alteration of the sense of taste, in its various manifestations, occurs in most cases in asymptomatic or paucisymptomatic patients—sometimes preceding the onset of full-blown disease [15] or occurring in an early phase of SARS-CoV-2 infection [73]. A clinical course analysis indicated that these patients may develop a variability of taste disturbances, both quantitative and qualitative ones: quantitative disturbances are classified in ageusia (complete loss of taste), hypogeusia (partial loss of gustatory sensitivity) and hypergeusia (increase of gustatory sensitivity); qualitative ones include dysgeusia (altered taste) divided in parageusia (altered perception of gustatory stimuli) and phantogeusia (taste impressions in the absence of stimulus) [74] (Figure 2). Symptoms usually occur within 3–5 days of the clinical onset of the disease [75]. In a prospective Italian multicentre study, ageusia or severe hypogeusia was present in 40.6% of patients within the first 4 days of the onset of COVID-19 symptoms [76]. However, gustatory dysfunctions can manifest even before diagnosis, as an onset symptom of SARS-CoV-2 infection (a study has shown this in 11% of cases [65]) or even as the only symptom [77].

An examination of Italian studies has indicated that the prevalence of gustatory symptoms can vary also based on the type of taste alteration with a prevalence of 8.5% for dysgeusia and 1.7% for ageusia [78]. Another Italian study showed that mild, moderate and severe hypogeusia, and ageusia are present in 22.2%, 15.3%, 9.7% and 1.4% of cases, respectively [75]. In a subsequent study by the same authors, the gustatory dysfunction was presented as follows: mild hypogeusia (prevalence of 24.2%), moderate hypogeusia (12.1%), severe hypogeusia (9.1%) and ageusia (6.1%) confirming the previous data on similar populations [79]. Two different Spanish studies found that the prevalence of ageusia, dysgeusia and hypogeusia was 45.2%, 25.8% and 22.6% in one [80]; in the other, a systematic review including 33 cross-sectional studies and 10,220 patients, found that the rate of prevalence of gustatory disturbances was 38% for dysgeusia, 35% for hypogeusia, 24% for ageusia, confirming the homogeneity of the results in populations belonging to the same ethnic group [34].

Taste dysfunction also seems to be related to the clinical course of SARS-CoV-2 infection disease. Initial studies carried out in China showed how taste alterations were associated with a milder course of coronavirus disease-19 [81], also confirmed by subsequent Italian studies in which it has been demonstrated that taste is more frequently altered in mild patients compared with severe ones [75]. Indeed, gustatory alterations have been described with prevalence of 78.9% in home care patients and of 51.9% in hospitalized patients [73]. These data are confirmed by several study conducted worldwide demonstrating a coherence and a common thought regarding this correlation [15].

Not only the absence or presence of the symptom but also the type of taste alteration has been correlated with the course of coronavirus disease-19 and proposed as a prognostic factor, an indicator of more or less severe forms of the disease. A recent study evidenced that umami, bitter and sweet tastes processing is more impaired in moderate patients than in mild ones and that these taste dysfunctions associated with parageusia could be considered spies of the more severe forms of COVID-19 [82].

The duration of gustatory disturbances is extremely variable, ranging from a short recovery time of impaired taste to a long-term deterioration. In subjects with rapid recovery, the average duration varied between 7 and 15 days based on the results of several studies ranging from a study that showed a recovery in 7.1 days [23] to an observation on Italian health workers of 16 days as the average duration of dysgeusia with a substantial difference between the two sexes (26 days for women, 14 for men) [83]. The severity of COVID-19 disease also seems to affect the duration of gustatory disturbances: in mild/moderate forms the average duration is shorter (7 days) than in severe/critical cases (11 days).

Some patients suffer from long-lasting impairment of smell and taste [84,85]; approximately 10% in a two-month follow-up and 11% in a 6-month follow-up showed no recovery [84] and only partial recovery was present in 30% of cases [85]. In some European and Asian cohort studies, dysgeusia persisted in 8% of cases after resolution of COVID-19 symptoms or 15 days after the negative RT-PCR test [86]. In a study across several European sites, 9.4% of patients reported dysgeusia at a mean follow-up of 63 days from the first report [87]. In an Italian study, patients with COVID-19 suffering from mild symptoms were followed from the first assistance control to four and eight weeks. The prevalence of gustatory disturbances was 60.1% at baseline, 36.6% compared to control at four weeks and 18.6% at the eighth week [88]. It has been shown that a fair number of patients present long-lasting gustatory impairments. It is also interesting to note that in COVID-19 subjects the dysfunction can occur simultaneously for taste and smell, and that for both these chemo-sensory systems, overlapping long-lasting times before healing are reported.

## 7. Conclusions

A high percentage of COVID-19 patients may have an altered sense of taste. This is often associated with olfactory disorders and occurs early in the course of the disease, sometimes being the only symptom. However, some distinctions must be made: (1) It is a symptom, in most cases collected with subjective methods such as surveys or self-reports; (2) other pathologies of the oral cavity, as well as the normal aging process can affect a change in the sense of taste. The patient’s medical history and a physical examination is therefore necessary to correlate this symptom to a SARS-CoV-2 infection. In this article, the term “hypothesis” occurs frequently; this is the logical consequence of a completely new virus and disease, for which diagnostics, pathogenesis and treatment are still largely “hypothetical”. Many studies are therefore still needed, not only for scientific speculation, but above all to better delineate the natural history of this infection.

## Data Availability

Not applicable.
